# Supplementary material for: A Crucial Role of Proteolysis in the Formation of Intracellular Dinitrosyl Iron Complexes
Source: Molecules. 2024 Apr 5;29(7):1630. doi: 10.3390/molecules29071630 (PMC11013114; doi:10.3390/molecules29071630)
Supplement: Supplementary file 1 [file molecules-29-01630-s001.zip › molecules-2878987-supplementary.pdf]

Supplementary figures

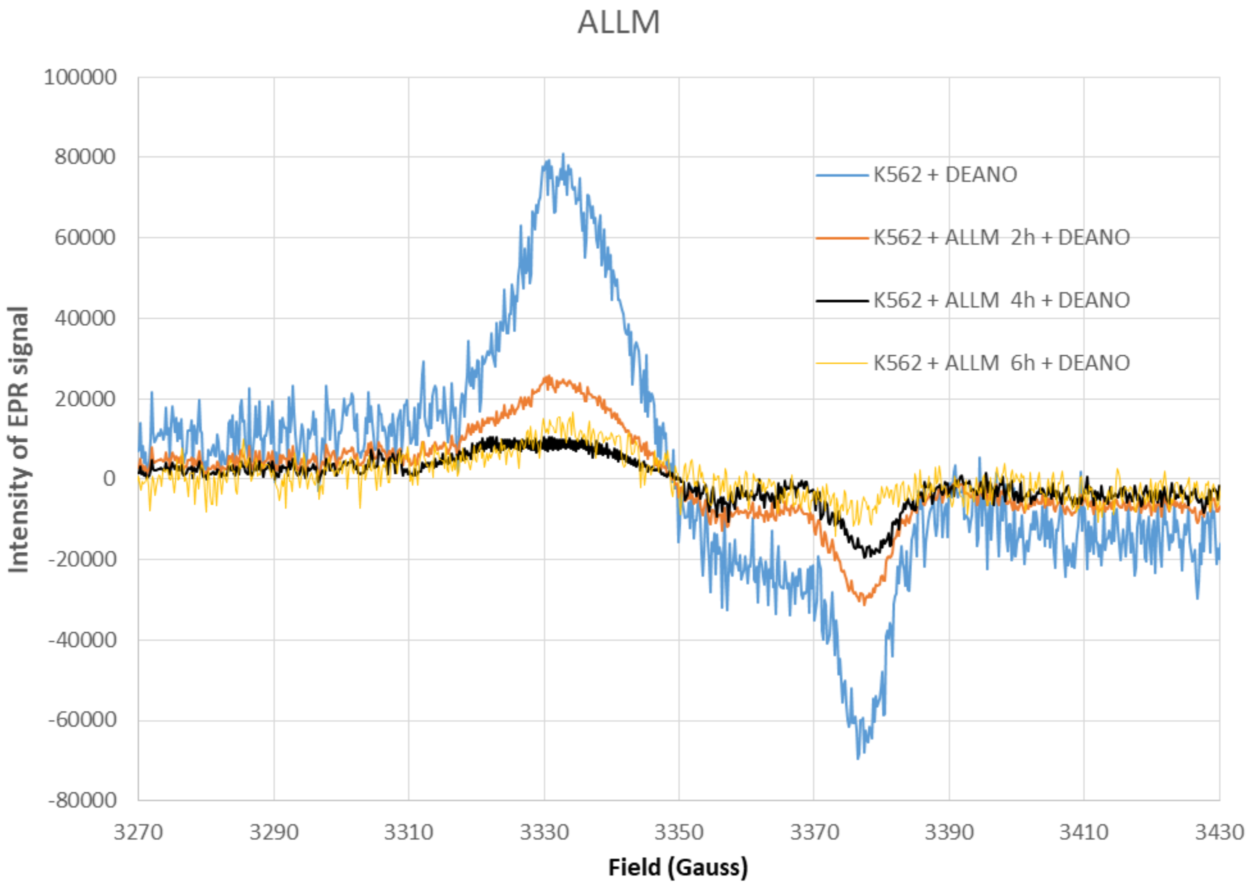

Supplementary Figure 1. Representative EPR spectra of K562 cells incubated with 50 µM ALLM for indicated time (0, 2, 4, 6 h) and then treated with 70 µM DEANO for 15 min at 37°C

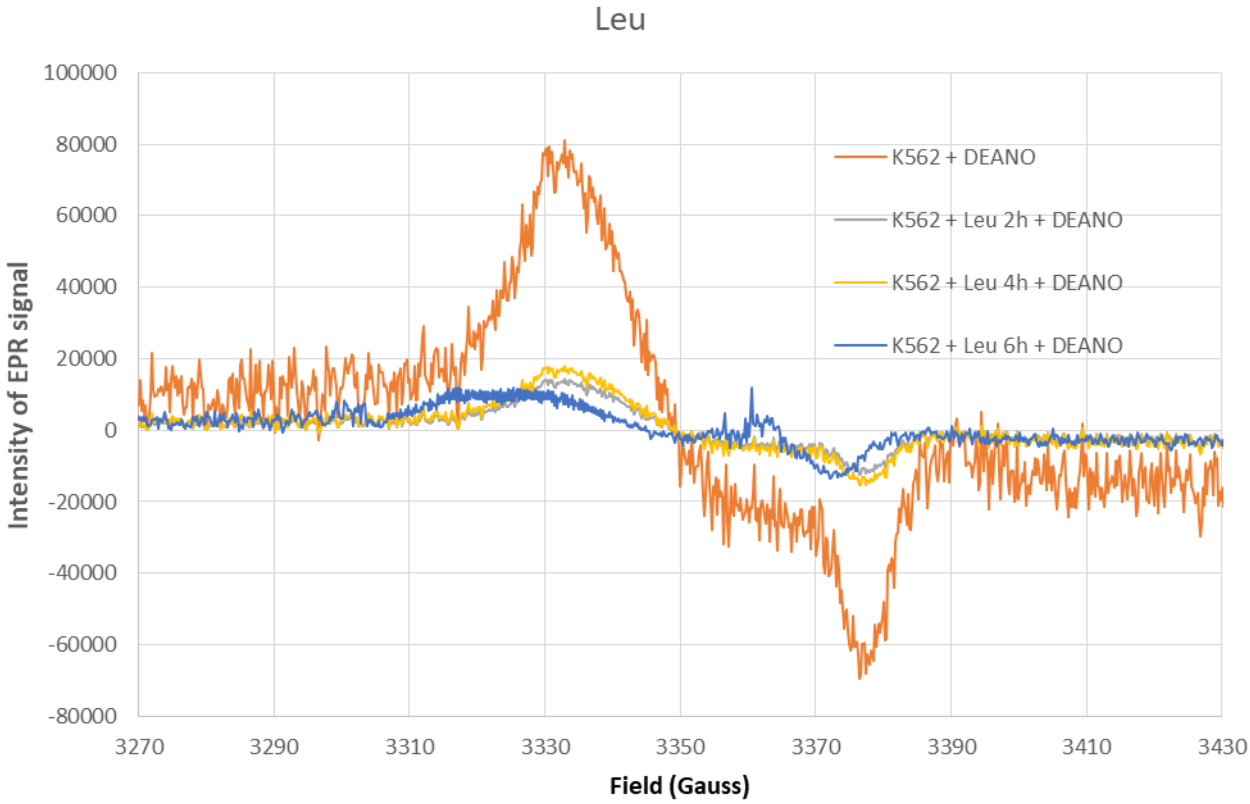

Supplementary Figure 2. Representative EPR spectra of K562 cells incubated with 100 µM leupeptin for indicated time (0, 2, 4, 6 h) and then treated with 70 µM DEANO for 15 min at 37°C

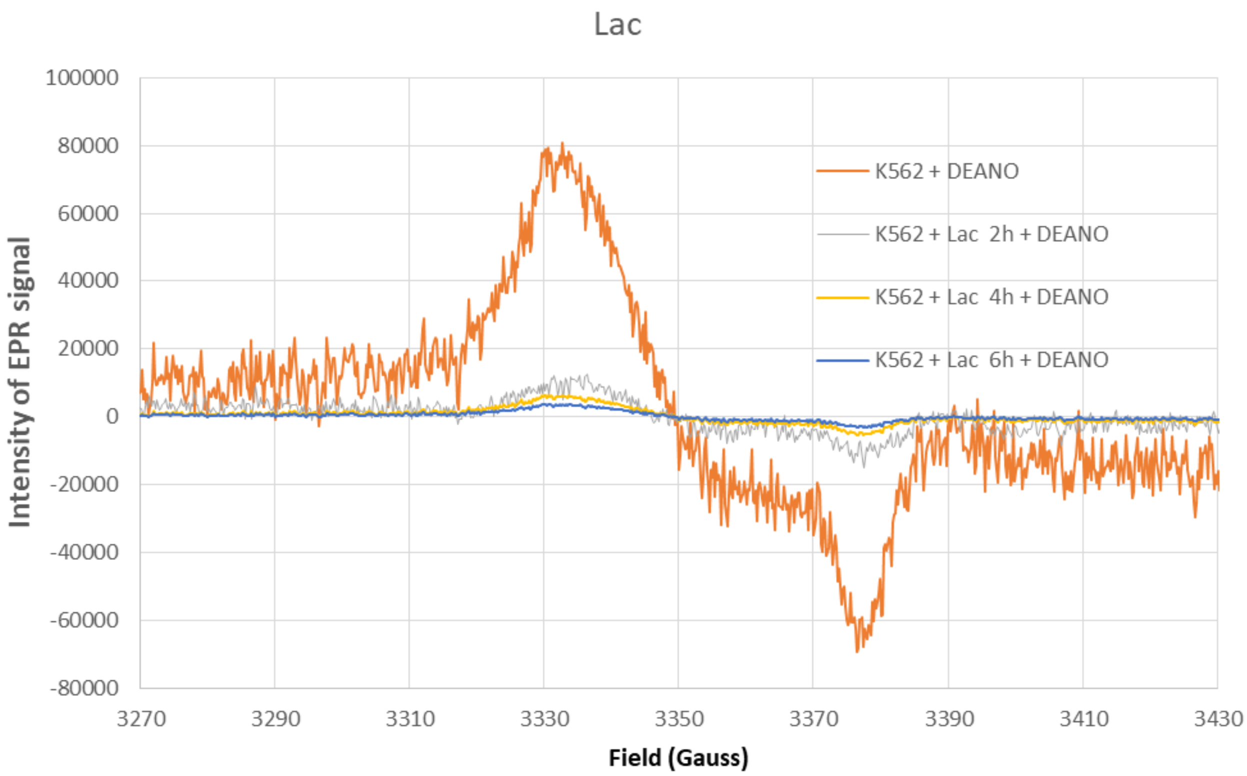

Supplementary Figure 3. Representative EPR spectra of K562 cells incubated with 20µM lactacystin for indicated time (0, 2, 4, 6 h) and then treated with 70 µM DEANO for 15 min at 37°C

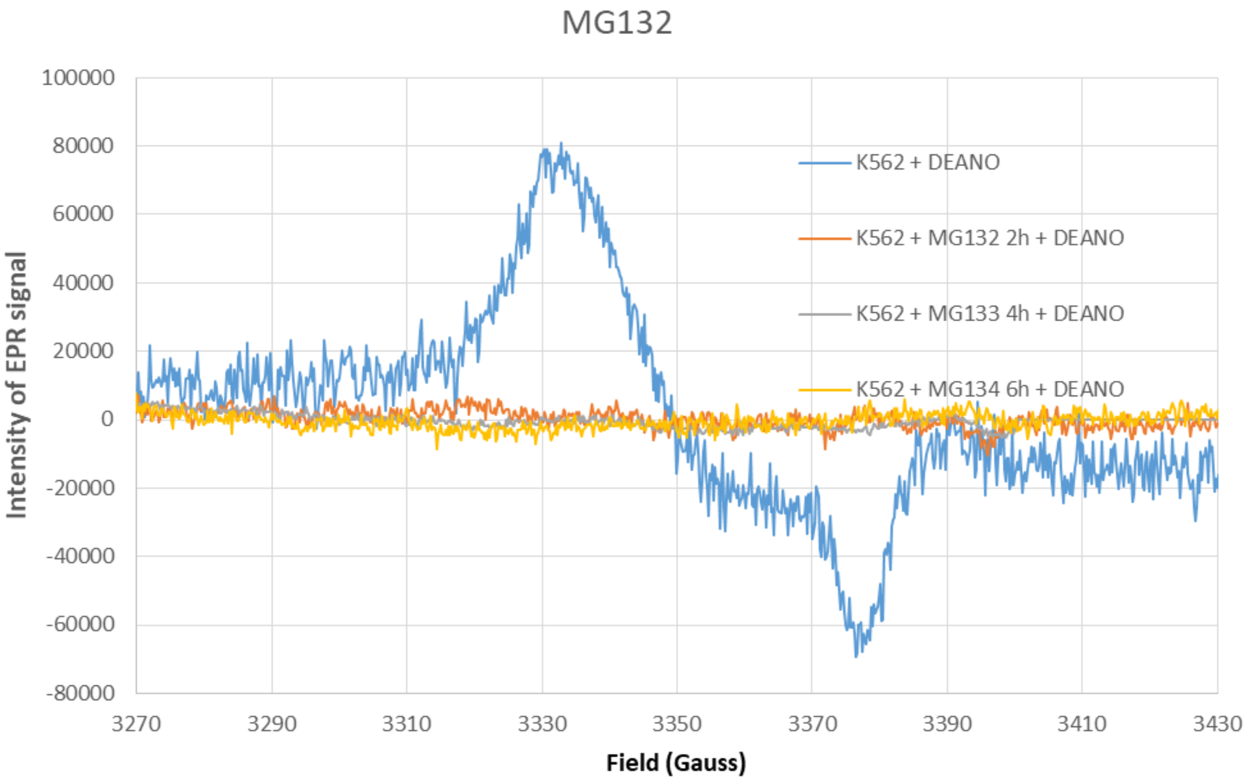

Supplementary Figure 4. Representative EPR spectra of K562 cells incubated with 25 µM MG132 for indicated time (0, 2, 4, 6 h) and then treated with 70 µM DEANO for 15 min at 37°C

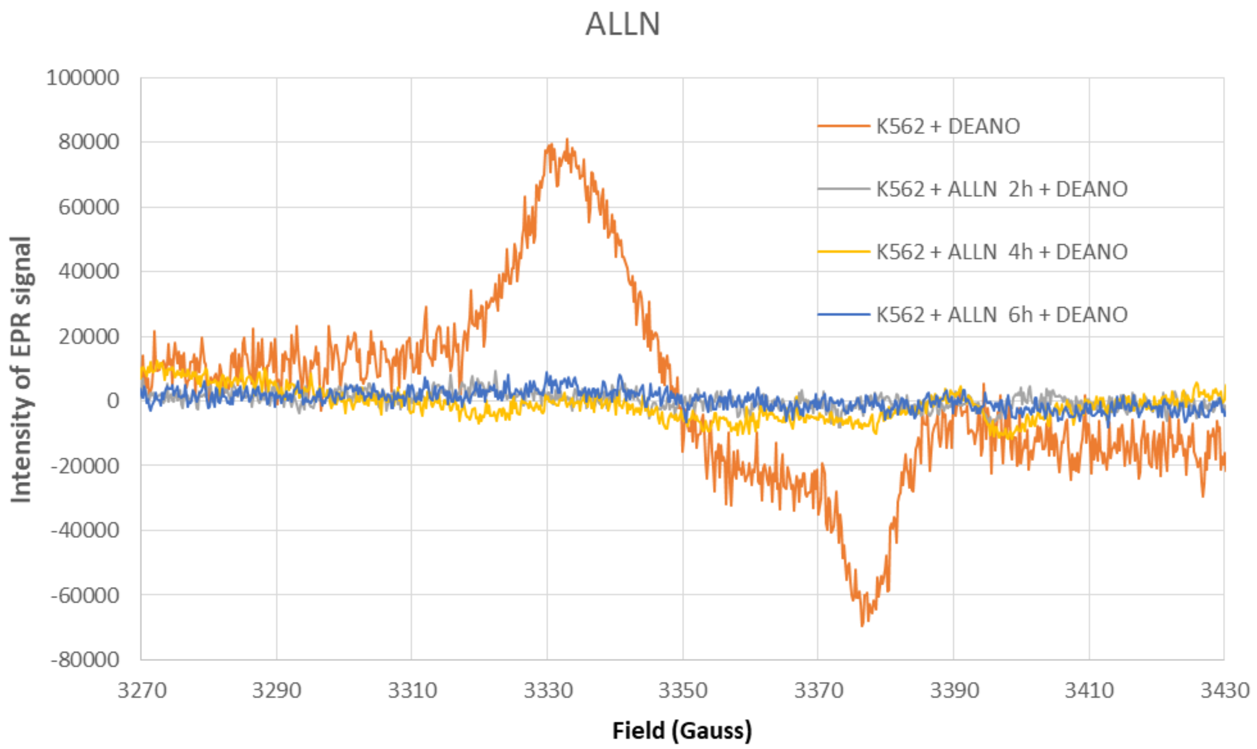

Supplementary Figure 5. Representative EPR spectra of K562 cells incubated with 20μM ALLN for indicated time (0, 2, 4, 6 h) and then treated with 70 μM DEANO for 15 min at 37°C
